# Epigenetic patterns associated with an ascidian invasion: a comparison of closely related clades in their native and introduced ranges

**DOI:** 10.1038/s41598-019-49813-7

**Published:** 2019-10-03

**Authors:** Nicola A. Hawes, Achira Amadoru, Louis A. Tremblay, Xavier Pochon, Brendon Dunphy, Andrew E. Fidler, Kirsty F. Smith

**Affiliations:** 1 Cawthron Institute, 98 Halifax Street East, Nelson, 7010 New Zealand; 2 Institute of Marine Science, University of Auckland, Private Bag 92019, Auckland, 1142 New Zealand; 3 School of Biological Sciences, University of Auckland, Private Bag 92019, Auckland, 1142 New Zealand

**Keywords:** Evolutionary ecology, Invasive species, DNA methylation, Molecular ecology

## Abstract

Environmentally induced epigenetic modifications have been proposed as one mechanism underlying rapid adaptive evolution of invasive species. *Didemnum vexillum* is an invasive colonial ascidian that has established in many coastal waters worldwide. Phylogenetic analyses have revealed that *D*. *vexillum* populations consist of two distinct clades; clade B appears to be restricted to the native range (Japan), whereas clade A is found in many regions throughout the world, including New Zealand. The spread of *D*. *vexillum* clade A suggests that it might be intrinsically more invasive than clade B, despite low levels of genetic diversity compared to populations from the native region. This study investigated whether *D*. *vexillum* clade A exhibits epigenetic signatures (specifically differences in DNA methylation) associated with invasiveness. Global DNA methylation patterns were significantly different between introduced clade A colonies, and both clades A and B in the native range. Introduced colonies also showed a significant reduction in DNA methylation levels, which could be a mechanism for increasing phenotypic plasticity. High levels of DNA methylation diversity were maintained in the introduced population, despite reduced levels of genetic diversity, which may allow invasive populations to respond quickly to changes in new environments. Epigenetic changes induced during the invasion process could provide a means for rapid adaptation despite low levels of genetic variation in introduced populations.

## Introduction

A major threat to biodiversity worldwide is the introduction, establishment and spread of invasive species beyond their native range^1,2^. Due to the considerable ecological and economic impacts of biological invasions^3^, research has focused on the prevention, control and eradication of invasive species^4^. Molecular-based approaches are often utilized to characterize the ecological and evolutionary factors involved in the invasion process^5–7^, and are effective for estimations of propagule pressure^8^, early detection of introduced populations^9^, identifying the source of introductions^10^, and identifying genetic characteristics associated with successful invaders^11^. A major goal of invasion biology is to understand why certain species are successful invaders in order to facilitate assessment and management of risks^7^.

Phenotypic plasticity, a wide tolerance to environmental stress, rapid growth and reproduction, and a high level of competitive ability have all been proposed to facilitate invasiveness^12^. Higher genetic diversity within populations is also thought to aid in the successful colonization of new environments^13,14^. Specifically, low levels of genetic variation are known to increase the risk of inbreeding depression and limit the adaptive and evolutionary potential of a species^12^. Yet, invasive populations with little or no genetic variation can be extremely successful in new environments^15^, sometimes outcompeting native species^16^. Reduced competition from conspecifics^17,18^, the maintenance of a high level of quantitative trait diversity^19^, and the release from natural enemies^20^ have been proposed as key factors determining invasion success when genetic diversity is low. More recently, epigenetic mechanisms (e.g., DNA methylation), which can increase both phenotypic plasticity and heritable variation, have been suggested to play a key role in invasion success^21–23^. Epigenetic mechanisms can alter a given genotype’s influence on an organism’s phenotype, without changes in the underlying DNA nucleotide sequences. By increasing, decreasing, or silencing the activity of genes, epigenetic changes can lead to novel phenotypic variation in the absence of additional genetic diversity^24^. Despite this, few studies have investigated epigenetic patterns in invasive populations, which is an important step in understanding the role of epigenetic modifications in successful biological invasions.

A valuable approach for identifying genotypes and phenotypes associated with successful invasive species is to compare populations in their native and non-native habitats^25,26^. Molecular analyses have revealed that invasive species are often comprised of distinct sibling species or clades across their expanded and native ranges^27–29^, and in some cases, only certain clades become invasive^18,25,30^. *Didemnum vexillum* is an invasive marine colonial ascidian that has become established in temperate coastal waters worldwide, including several regions in New Zealand^31,32^, North America^33^, and Europe^34–36^. *D. vexillum* is believed to have been translocated throughout the world as fouling on aquaculture species and equipment, such as cultured Pacific oysters (*Crassostrea gigas*), and on ship hulls^37–39^. Additional to this well-defined history of spread, morphological^37^ and molecular comparisons^18,40,41^ have concluded that the native range of *D*. *vexillum* is likely to be the Northwest Pacific Ocean, including Japan. Phylogenetic analyses have revealed that *D*. *vexillum* populations consist of two distinct clades^18^. One clade (referred to as clade B) appears to be geographically restricted to *D*. *vexillum’s* native region (Northwest Pacific Ocean), whereas colonies belonging to clade A have become established in temperate coastal areas around the world, including New Zealand. Clades A and B are morphologically identical^37^, and the level of the mitochondrial cytochrome *c* oxidase subunit I (COI) gene sequence divergence between the two clades is far below the comparable divergence values for other ascidian species, such as the recently classified cryptic species *Ciona robusta* and *Ciona intestinalis* (formally *C*. *intestinalis* type A and type B, respectively)^26,42–44^. Therefore, evidence to date suggests that *D*. *vexillum* clades A and B represent an ongoing speciation event^18,26^. Diversification of clades A and B within the Northwest Pacific following a human-mediated introduction from another region is not consistent with known evolutionary rates at COI, as divergence of the A and B COI clade lineages is estimated to have occurred 1.5–2.5 million years ago, which pre-dates anthropogenic vectors^18^. The persistence of clade B’s restricted distribution may indicate that it is intrinsically less invasive than clade A^18^. Introduced populations of clade A in New Zealand have low levels of genetic diversity compared to populations within the native region of Japan, indicating a founder effect^18^. Despite this, introduced *D. vexillum* populations have been extremely successful in their new environments, forming large colonies that can extend over significant areas, including commercially important aquaculture farms^39^.

The most well-studied epigenetic mechanism related to invasion success is DNA methylation, which generally refers to the addition of a methyl group to cytosine nucleotides to form 5-methylcytosine. DNA methylation can respond to environmental cues^45–47^, and has been associated with adaptation to different environmental conditions in invaded habitats^48,49^. Additionally, some introduced populations have higher DNA methylation diversity than populations in their native range despite reductions in genetic diversity due to founder effects^50^, and lower levels of global or genome-wide DNA methylation^51^, both of which have been associated with phenotypic variation and phenotypic plasticity^51–54^. Given the tendency of *D*. *vexillum* clade A to successfully invade multiple regions throughout the world, we hypothesized that (1) introduced populations of *D*. *vexillum* clade A would have high levels of DNA methylation diversity, despite low genetic diversity, (2) introduced populations of *D*. *vexillum* clade A would have lower levels of global DNA methylation compared to populations within the native region, and (3) populations would be differentially methylated when collected from sites with different environmental conditions. To test these hypotheses, we compared whole genome DNA methylation in *D*. *vexillum* colonies collected from New Zealand (clade A) with those from Japan (clade A and clade B), and between sites with different environmental conditions (differences in temperature and salinity). Samples used in the present study were a subset of samples previously collected and analysed by Smith, *et al*.^18^ using mitochondrial cytochrome *c* oxidase subunit I (COI) partial coding sequences.

## Materials and Methods

### Tissue sample collection

**Figure 1:**
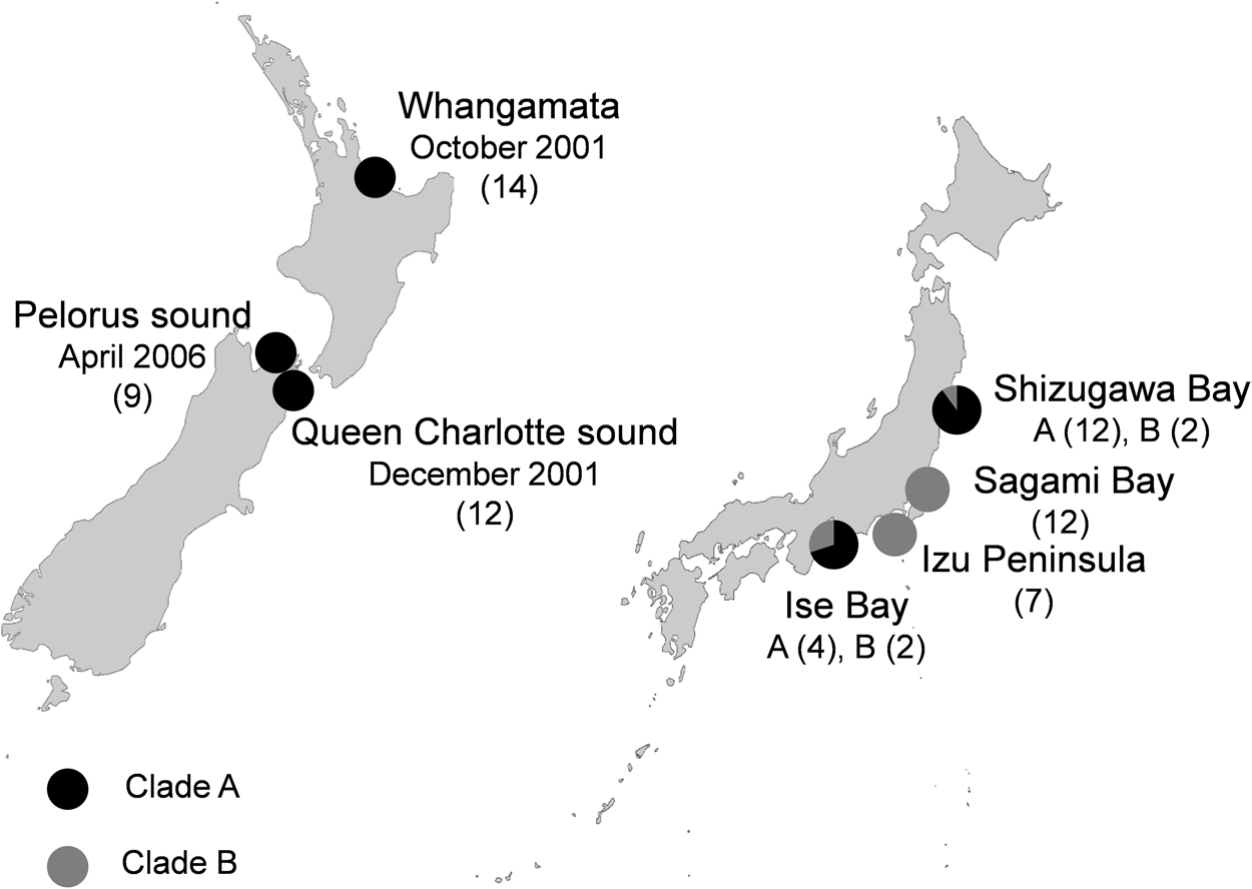
Locations and details of *Didemnum vexillum* colonies used for analyses. Invasive populations in New Zealand (left) consist of clade A colonies only, whereas populations from within the native range of Japan (right) are comprised of both clade A and clade B colonies. Pie charts represent sampling sites used for mitochondrial cytochrome *c* oxidase subunit I (COI) gene analysis in Smith *et al*.^18^, and the relative proportions of each clade. Labelled sites represent the location of samples selected for DNA methylation analysis in this study. Figure adapted from Smith *et al*.^18^. The date *D*. *vexillum* populations were first recorded in each location in New Zealand is indicated below each site and the number of colonies sampled is indicated in brackets.

*Didemnum vexillum* tissue samples were collected between April 2008 and July 2009 from four sites in Japan (n = 39) and three sites in New Zealand (n = 35) by Smith, *et al*.^18^. Samples were collected in the summer months of the respective countries. Tissue samples were preserved in approximately 2.0 ml of 96% (v/v) ethanol and stored at -20 °C before being assigned to clade A or clade B by mitochondrial COI coding region analysis^18^. All colonies from New Zealand were clade A (n = 35), whereas samples collected from Japan were comprised of clade A (n = 16) and clade B (n = 23). In Japan, the distributions of clades A and B overlap at several sites (Fig. 1). The earliest worldwide record of *D. vexillum* was in Japan in 1926, but it was only identified as *Didemnum* sp. at the time^37^. In New Zealand, *D*. *vexillum* was first reported in 2001 in the North Island, in Whangamata Harbour^31^ and was described initially as a native species to New Zealand. Two months later, *D*. *vexillum* was discovered in the South Island, in Queen Charlotte Sound^55^. It is thought to have been transported to the South Island as fouling the hull of a large barge that had earlier been anchored in Whangamata Harbour^55^. Due to the continued movement of vessels, *D*. *vexillum* subsequently spread throughout the Marlborough Sounds, including to Pelorus Sound^39^.

### Environmental conditions

Site-specific environmental data (temperature and salinity) were collated from publicly available reports and websites (Table 1). Based on experiments identifying *D*. *vexillum* tolerance limits and the optimal temperature and salinity range for growth^46^, we allocated a temperature and salinity ‘stress score’ to each site, with 1 being the lowest stress score and 3 being the highest (Table 2). Temperature and salinity stress scores related to tolerance ranges were as follows; Temperature stress: *D*. *vexillum* grows well in temperatures less than 23 °C, so a stress score of 1 was allocated to sites with maximum temperatures of <23 °C, *D*. *vexillum* growth is usually reduced at temperatures higher than 24 °C, so sites with a maximum temperature of 23–25 °C were given a stress score of 2, growth is further reduced above 25 °C, and 28 °C is close to the upper tolerance limit for *D*. *vexillum*^46,56^, so sites with a maximum summer temperature of 25–28 °C were given a stress score of 3; Salinity stress: *D*. *vexillum* can grow well in sanities above 26 PSU^46^, so sites with minimum salinity values ≥26 were given a stress score of 1, growth is generally reduced below 26 PSU, and ascidians are rarely found in salinities lower than 25 PSU^57^, so a stress score of 2 was given to sites with minimum salinity values of 23–<26 PSU, a stress score of 3 was allocated to sites that drop below 23 PSU, as 20 PSU is thought to be close to the low salinity limit for *D*. *vexillum*, with extended periods of exposure resulting in mortality^58,59^. We then combined the temperature and salinity stress score at each site to obtain a ‘summative stress score’ which was used for further analysis (see Table 2). This is a similar approach to the method described in Ardura *et al*.^60^.

**Table 1:** Geographical coordinates and general environmental conditions (temperature and salinity) at the sample collection sites in New Zealand (NZ) and Japan (JA).

Site of collection	Sample code	Clades present	Geographical coordinates	Temperature mean (min–max)	Temperature range (min–max)	Salinity mean (min–max)	Salinity (min–max)
**New Zealand**	**NZ**						
Whangamata Harbour	Wha	A	37°11′52.3″S 175°51′58.6″E	13.5–19.5	12.5–23	25–34	13–35.5
Pelorus Sound	Pel	A	41°09′46.1″S 173°51′48.5″E	12–18.5	11.5–20.5	33–35	23–35
Queen Charlotte Sound	Que	A	41°15′00.0″S 174°00′34.3″E	11–17	11–19	34–35	30.5–35
**Japan**	**JA**						
Shizugawa Bay	Shi	A, B	38°39′21.8″N 141°29′05.2″E	8.5–22	6–23.5	33–34	29.5–34
Ise Bay	Ise	A, B	34°44′01.6″N 136°43′10.1″E	11–25	9–27	29–32	21–33
Izu Peninsula	Izu	B	34°39′56.3″N 138°56′23.5″E	13.5–24.5	10.5–26	31–33	27–33
Sagami Bay	Sag	B	35°14′01.0″N 139°22′42.7″E	12–26	10–28	NA	34–35

Temperature range is reported in °C, salinity is reported in practical salinity units (PSU). Site-specific environmental data (temperature and salinity) were collated from available reports and websites as follows: Whangamata Harbour (Environment Waikato Regional Council); Pelorus Sound and Queen Charlotte Sound (National Institute of Water and Atmospheric Research, Water Quality in the Marlborough Sounds Annual Monitoring report July 2014–2015 prepared for Marlborough District Council, and https://cawthron.shinyapps.io/WQ-Marlborough/); Shizugawa Bay (Shizugawa Nature Centre and Takahashi *et al*.^95^); Ise Bay (Sugashima Marine Biological Laboratory, Nagoya University); Izu Peninsula (Shimoda Marine Research Centre, University of Tsukuba http://www.shimoda.tsukuba.ac.jp/kansoku.html); Sagami Bay (The University of Tokyo Department of Biological Sciences Annual Report, 2008 and Sogawa *et al*.^89^).

**Table 2:** Site-specific stress scores related to *Didemnum vexillum* tolerance ranges based on method described in Ardura *et al*.^60^.

Site of collection	Sample code	Clades present	Temperature score	Salinity score	Summative score
**New Zealand**					
Whangamata Harbour	Wha	A	1	3	4
Pelorus Sound	Pel	A	1	2	3
Queen Charlotte Sound	Que	A	1	1	2
**Japan**					
Shizugawa Bay	Shi	A	2	1	3
Ise Bay	Ise	A	3	3	6
Izu Peninsula	Izu	B	3	1	4
Sagami Bay	Sag	B	3	1	4
Shizugawa Bay	Shi	B	2	1	3
Ise Bay	Ise	B	3	3	6

Summative score = temperature score + salinity score.

The sampling sites represent different environments within both New Zealand and Japan (Table 1). New Zealand seawater temperatures are similar between sites and within the optimal range for *D*. *vexillum*, with winter minima around 11–12 °C, and maxima between 19 °C and 23 °C. Therefore, we allocated all New Zealand sites a temperature stress score of 1 (Table 2). While the mean and maximum salinity values are similar between sites in New Zealand (mean approx. 33–35 PSU), the minimum salinity is more variable, with salinity above 30 PSU in Queen Charlotte Sound but dropping below 25 PSU in Pelorus Sound and Whangamata Harbour. The lowest salinity values were reported in Whangamata (13 PSU). Minimum and maximum temperatures and salinities are most likely to influence global DNA methylation differences (see Hawes *et al*.^46^). Therefore, each site in New Zealand was allocated a different salinity stress score based on minimum salinity values (1, 2 and 3 respectively; Table 2).

For Japan, minimum water temperatures were similar, or slightly cooler, than minimum water temperatures at sites in New Zealand. Minimum temperatures ranged from 9–12.5 °C at all sites, except for Shizugawa Bay which can be <7 °C in winter. The maximum temperature in Shizugawa Bay was slightly warmer than the maximum water temperatures at New Zealand sites (19–23.5 °C), while Sagami Bay, Ise Bay, and Izu Peninsular all reach maximum water temperatures of 26–28 °C (Table 1). Therefore, all sites in Japan were allocated stress scores of 2 or 3 (Table 2). For salinity, Shizugawa Bay salinity values were most similar to Queen Charlotte Sound, while Ise Bay salinity values were lower, and more similar to Pelorus Sound and Whangamata Harbour. Major rivers empty into Pelorus Sound (Pelorus River), Whangamata Harbour (Wentworth River) and Ise Bay (Kiso Three Rivers) which can result in low minimum salinity values following precipitation events, so these sites were given stress scores of 2 or 3 while all other sites obtained a salinity stress score of 1 (Table 2).

### DNA extraction and methylation-sensitive amplified polymorphism

Tissue samples were finely chopped up using a sterile scalpel blade and genomic DNA was obtained using G-spin Total DNA extraction kits (animal tissue protocol; Intron, Gyeonggi-do, South Korea). Methylation-sensitive amplified polymorphism (MSAP) was then used to determine changes in global methylation patterns as previously described in detail in Hawes *et al*.^46^.

### Data analysis

The Peak Scanner software (Applied Biosystems, Foster City, CA, USA) was used to assign the MSAP fragments peak height and size. To establish the peak height with the lowest error rate (the lowest number of fragment presence/absence mismatches between triplicate samples), and the error rate for *msap* (v. 1.1.8) analysis^61^ described below, we completed the MSAP procedure described above in triplicate for one individual sample. The following settings were associated with the lowest error rate between triplicates: error rate per primer, 0.07; analysis range, 50–500 base pairs (bp); minimum peak height, 50 relative fluorescent units (RFU). Peak presence/absence data corresponding to *Hpa*II and *Msp*I fragments was then converted to a binary matrix (presence = 1, absence = 0), so that the methylation state of each restriction site could be identified. The MSAP profiles were then assessed in R^62^ using the *msap* package (v. 1.1.8)^61^.

The *msap* package determines whether individual fragments (loci) are methylation-susceptible loci (MSL) or non-methylated loci (NML) by analyzing the contents of the binary matrix. Subsequent analyses are performed independently, with MSL used to assess DNA methylation variation and NML used to assess genetic variation^61^. While NML has been used to assess genetic variation in several studies^51,60,63^, it is only a proxy for genetic variation and should be interpreted with some caution^64^. Methylation state is categorized as either: Type I = unmethylated state, Type II = methylation of internal cytosine, Type III = hemimethylation, and Type IV, which is considered uninformative as this type could be due to either hypermethylation or the absence of the target restriction site^61,65^. Within *msap*, epigenetic (MSL) and genetic (NML) differentiation between populations were assessed by principal coordinates analyses (PCoA) followed by analyses of molecular variance AMOVA^66^ with 10000 permutations^61^. A Mantel test was used to establish the correlation between MSL and NML, an indication of how much epigenetic variation might be influenced by the genetic background^61^. The amount of genetic and epigenetic variation was estimated using the Shannon diversity index (S). Differences between NML and MSL diversity were tested using the Wilcoxon Rank Sum test with continuity correction.

Following analysis with *msap*, the overall MSL and NML variation estimated by the Shannon diversity index was compared between populations using the number of samples (N), mean diversity (I) and the standard deviation (obtained from *msap*) to perform and one-way analysis of variance (ANOVA) and post-hoc pairwise comparisons using Tukey’s honest significance difference test (Tukey’s HSD). The global methylation level was calculated as the proportion of methylated loci (the number of methylated loci (Types II and III) divided by the scorable loci (Types I, II and III)) following^51,67^. Using R (v. 3.3.0)^62^, global methylation levels were compared among populations using a generalised linear model (GLM) with a quasibinomial distribution to correct for overdispersion. Residuals were checked and assumptions validated. We then compared global methylation differentiation in colonies collected from sites with the same summative stress scores using *msap* (v. 1.1.8)^61^ (PCoA followed by AMOVA) to determine whether site-specific MSL differences were associated with differences in environmental conditions (temperature and salinity). Only summative stress scores ‘3’ and ‘4’ could be analysed with *msap* because scores ‘2’ and ‘6’ were only associated with one site respectively. To assess the relationship between global methylation levels and environmental conditions, we used a GLM with a quasibinomial distribution to compare the proportion of methylated loci in colonies collected from sites with different summative stress scores, followed by post-hoc pairwise comparisons using Tukey’s HSD tests.

## Results

### Environmental differences between sites

DNA methylation patterns in colonies from sites with the same summative stress scores were significantly differentiated (Supplementary Table S1), suggesting that global DNA methylation differences between sites in this study are not clearly associated with summative temperature and salinity scores. There were also no significant differences between the proportion of methylated loci (Types II and III/Types I, II and III) associated with different summative stress scores (Supplementary Table S2).

### DNA methylation and genetic differentiation

**Figure 2:**
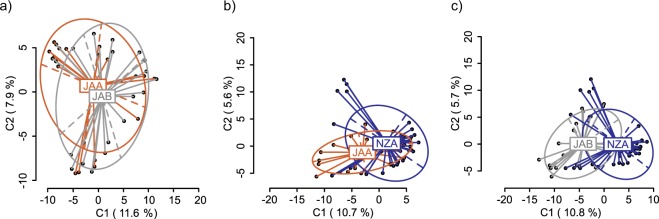
Principal coordinates analyses (PCoA) showing: (**a**) non-significant methylation (MSL) differences between *Didemnum vexillum* colonies from Japan clade A (JAA) and Japan clade B (JAB), (**b**) significant MSL differences between colonies from Japan clade A (JAA) and New Zealand clade A (NZA) and (**c**) significant MSL differences between colonies from Japan clade B (JAB) and New Zealand clade A (NZA). The first two coordinates (C1 and C2) are shown with the percentage of variance explained by them. Points in each group cloud represent individuals from different groups. Country and clade labels show the centroid for the points cloud in each group. Ellipses represent average dispersion of those points around their centre^61^. AMOVA tests for MSL differences are shown in Table 3. NZA (n = 35), JAA (n = 16) and JAB (n = 23).

In New Zealand colonies (clade A), 1784 loci were identified, 1309 of these were MSL (73.4%, 94% polymorphic) and 475 were NML (26.6%, 97% polymorphic). In Japan clade A, 1780 loci were identified, 1496 of these were MSL (84%, 92% polymorphic), and 284 were NML (16.6%, 97% polymorphic). Finally, in Japan clade B, 1785 loci were identified, 1658 were MSL (92.9%, 93% polymorphic) and 127 were NML (7.1%, 96% polymorphic). Combined, 1799 loci were identified. 1617 were MSL (89%, 99% polymorphic) and 182 were NML (10%, 99% polymorphic). We found no significant differences in MSL or NML patterns between Japan clade A (JAA) and Japan clade B (JAB). Instead, significant differences were due to colonies collected in New Zealand being different to colonies collected in Japan, regardless of clade affiliation (Table 3 and Fig. 2). When clades collected in Japan (JAA and JAB) were compared, the methylation patterns almost fully overlapped (Fig. 2a). However, when colonies collected in New Zealand (NZA) and Japan (JAA and JAB) were compared, they were partially differentiated, regardless of clade (Fig. 2b,c). MSL differences between NZA and JAA were due to colonies collected from Pelorus (PelA) and Whangamata (WhaA) (New Zealand) being different to colonies collected from Japan (Shizugawa (ShiA) and Ise (IseA)). In contrast, there were no significant MSL differences between colonies collected from Queen Charlotte, New Zealand (QueA) and clade A colonies collected from Japan (Supplementary Table S3, MSL). For NML, there were significant differences between NZA and JAA at all sites, except for QueA and ShiA (Supplementary Table S3, NML). MSL differences between NZA and JAB were due to colonies collected from Izu (IzuB) and Sagami (SagB) being different to colonies from all sites in New Zealand (PelA, WhaA, and QueA) (Supplementary Table S4, MSL). For NML, there were significant differences between JAB and NZA colonies from all sites, with the exception of WhaA and QueA (NZ) which were not significantly different from ShiB (JA) (Supplementary Table S4, NML).

**Table 3:** Analysis of molecular variance (AMOVA) and degrees of freedom (d.f.) for methylation-sensitive loci (MSL) and non-methylated loci (NML) in *Didemnum vexillum* clade A colonies from Japan (JAA) and clade B colonies from Japan (JAB), clade A colonies from New Zealand (NZA) and clade A colonies from Japan (JAA), and clade A colonies from New Zealand (NZA) and clade B colonies from Japan (JAB). Significant p-values are shown in bold (*p* < 0.05).

Populations	Source	d.f.	SSD	MSD	Variance	Phi_ST	*p* value
**MSL**							
JAA - JAB	Among groups	1	364.9	364.9	−0.3955	−0.00106	0.4573
	Within groups	37	13778	372.4	372.4		
	Total	38	14143	372.2			
NZA - JAA	Among groups	1	571.3	571.3	11.75	0.03616	**0**.**0011**
	Within groups	49	15349	313.3	313.3		
	Total	50	15921	318.4			
NZA - JAB	Among groups	1	1061	1061	26.5	0.07538	**0**.**0001**
	Within groups	56	18205	325.1	325.1		
	Total	57	19266	338			
**NML**							
JAA - JAB	Among groups	1	23.39	23.39	0.3062	0.0171	0.0541
	Within groups	37	651.4	17.61	17.61		
	Total	38	674.8	17.76			
NZA - JAA	Among groups	1	82.63	82.63	2.22	0.06149	**0**.**0018**
	Within groups	49	1660	33.88	33.88		
	Total	50	1743	34.86			
NZA - JAB	Among groups	1	97.43	97.43	2.672	0.1031	**0**.**0001**
	Within groups	56	1302	23.25	23.25		
	Total	57	1400	24.56

There were no significant MSL or NML differences between colonies collected from different sites within Japan (MSL ɸ_ST_ = 0.01559, *p* = 0.1072, Supplementary Fig. S5; NML ɸ_ST_ = −0.01433, *p* = 0.7505). In contrast, there were significant differences between all sites in New Zealand (MSL, ɸ_ST_ = 0.05517, *p* =  < 0.001, Supplementary Fig. S5; NML ɸ_ST_ = 0.07178, *p* = 0.0033, Supplementary Table S6). NML and MSL were significantly positively correlated (Mantel test; JAA, r = 0.725, *p* = <0.001; JAB, r = 0.368, *p* = <0.001; NZA, r = 0.746, *p* = <0.001).

### DNA methylation and genetic diversity

**Figure 3:**
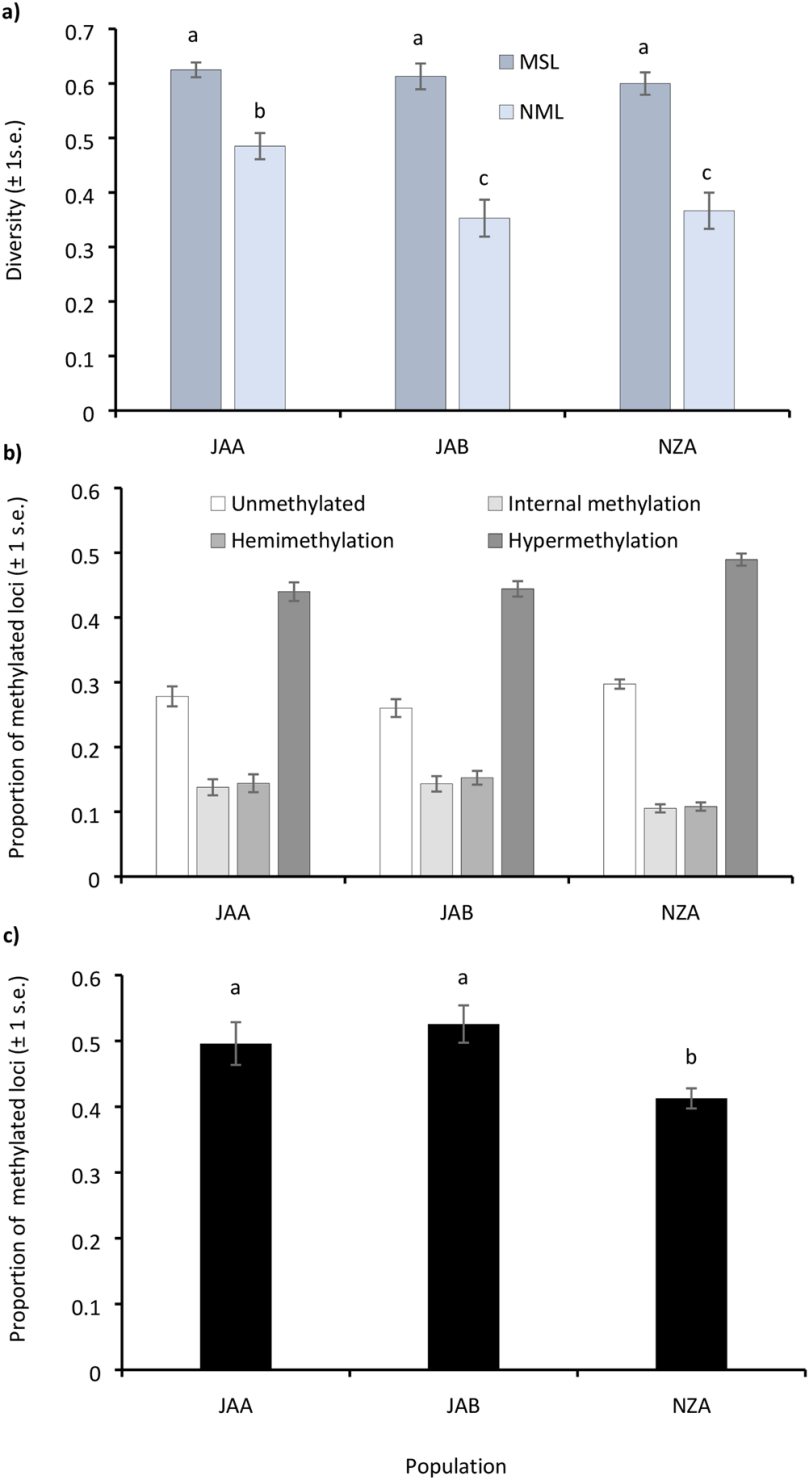
Bar graphs showing MSL and NML diversity and global DNA methylation levels in *Didemnum vexillum* colonies collected from Japan clade A (JAA, n = 16), Japan clade B (JAB, n = 23) and New Zealand clade A (NZA, n = 35): (**a**) DNA methylation (MSL) and genetic (NML) diversity; (**b**) methylation-susceptible loci classified as: unmethylated (Type I), internal methylation (Type II), hemimethylation (Type III), and hypermethylation or absence of restriction site (Type IV); and (**c**) global methylation levels measured as the proportion of methylated loci (Type II + III/scorable loci (Types I + II + III)) following Nicotra *et al*.^67^ and Ardura *et al*.^51^. Significant differences between treatments are denoted by different letters (*p* < 0.05).

MSL diversity was similar in all three populations (JAA, JAB, and NZA), and DNA methylation diversity (MSL) was significantly higher than genetic diversity (NML) (Fig. 3a and Supplementary Table S7). In contrast, there were significant differences in NML diversity between populations (Supplementary Table S8). NML diversity was significantly higher in JAA compared to both JAB and NZA (Supplementary Table S9).

### DNA methylation levels

The proportion of MSL classified as unmethylated, internally methylated, hemimethylated or hypermethylated for JAA, JAB, and NZA is shown in Fig. 3b. In all three groups, a greater proportion of Type IV methylation (hypermethylation) was detected compared to unmethylated or methylated loci. However, as Type IV methylation can also be due to absence of the restriction target (mutation) it is considered uninformative and is not used to calculate the proportion of methylated loci^51,60^. For the proportion of methylated loci, recently introduced New Zealand colonies had significantly lower methylation levels than colonies collected from within the native region (Japan) (*p* < 0.05, Supplementary Table S10). For JAA, JAB and NZA, the methylated loci (Types II and III) were 49%, 53% and 41% of the scoreable loci respectively (Fig. 3c). There were no significant differences in the proportion of methylated loci between JAA and JAB.

## Discussion

Comparing closely related clades, only one of which has become invasive, provides a unique opportunity to identify genotypes and phenotypes that might be associated with invasion success^26^. Significant global DNA methylation (MSL) and genetic (NML) differences were detected between *Didemnum vexillum* colonies introduced to New Zealand, and both clades in the native range (Japan). These differences were evident when looking at both differentiation and global DNA methylation levels, which will be discussed in detail below. In contrast, we found no significant differences in MSL or NML patterns between *D*. *vexillum* clades A and B collected from within the native range, despite the predicted divergence of *D*. *vexillum* COI clade lineages being between 12,000 and 2.6 million years ago^18^. A recent study of genome-wide DNA methylation variation within and between five house sparrow subspecies in their native range (Middle East, Iranian plateau) also found the majority of subspecies were not significantly differentiated, despite also inhabiting different environmental conditions (e.g., arid desert, humid forest, tropical coast). Most of the observed DNA methylation variation could be attributed to differences within subspecies rather than among subspecies, and appeared to follow a similar pattern to genetic differentiation among populations, which was also very low^68^. In contrast, a recent study of global DNA methylation in co-occurring subspecies of the facultatively clonal plant, *P*. *australis*, found significant differentiation of DNA methylation patterns between subspecies that inhabit the same environment^69^. However, these subspecies have very different life histories; one subspecies is native and the other introduced. Therefore, epigenetic differences between these subspecies might be associated with changes induced by invasion history^69^. In *D*. *vexillum*, the level of COI sequence variation between clades A and B (as estimated by percentage of polymorphic sites) is low compared to values reported for other ascidian species^18^, and colonies from the two clades are morphologically identical and occur in sympatry at several sites in Japan^37^. The lack of global DNA methylation differentiation between clades A and B in Japan might, therefore, represent similar genetic and life history backgrounds.

Clade A colonies collected from Japan had significantly higher levels of genetic (NML) diversity compared to the two other populations studied (NZA and JAB). While differences in genetic variation do not necessarily reflect differences in quantitative traits, it has been theorized that a high level of standing genetic variation will increase adaptive and evolutionary potential, which may facilitate the colonisation of new environments^70^. High levels of genetic variation within the native range (as found in JAA) may facilitate the spread of invasive species like *D*. *vexillum*. In the copepod, *Eurytemora affinis*, two clades have overlapping distributions in several estuaries and salt marshes, but one clade has invaded freshwater habitats while the other remains restricted to the native range^25^. The invasive clade was found to have higher DNA nucleotide diversity than the non-invasive clade within the native range^25^, and has evolved increased haemolymph osmolality following the invasion of freshwater habitats^71,72^. Comparisons of geographically restricted and widespread species also suggest that higher levels of genetic variation in ecologically important traits contribute to larger niche breadth^73,74^. While it is possible that future sampling might reveal that clade B has also invaded other regions, the rate of discovery suggests that if it is present, it is rare. Clade A has been identified in five broad sampling areas (New Zealand, Japan, West Coast North America, East Coast North America and Europe), whereas clade B haplotypes have been exclusively amplified from Japan^18^. It is also possible that clade A is only invasive ‘by chance’. Clade A and clade B haplotypes are not equally abundant nor equally distributed among sites in Japan^41^, and the probability of a haplotype being transported during an invasion event is proportional to the frequency of that haplotype in the native population where the transport vector originated^75^. This effect would be amplified if introductions to new regions were secondary invasion events rather than multiple primary introductions originating from the native range^41^.

Genetic variation based on NML should be interpreted with caution^64^, but lower genetic diversity in NZA is consistent with previous studies that have concluded that introduced populations of *D*. *vexillum* have experienced a reduction in genetic diversity, indicating a founder effect^18^. If lower levels of genetic diversity are in fact limiting adaptive potential, alternative mechanisms for increasing phenotypic variation, such as epigenetic variation, might be especially important during the introduction and establishment phases. Despite differences in genetic diversity, there were no significant differences in DNA methylation diversity between NZA, JAA, and JAB. A similar result was found when DNA methylation diversity was compared across house sparrow populations with varying invasion histories^53^. Combined, these results support the idea that epigenetic variation could preserve the ability of invasive populations to adapt following reductions in genetic diversity that can be associated with colonisation^53,54^, although the functional significance of epigenetic diversity remains unknown.

DNA methylation patterns were significantly differentiated between sites in the introduced range of *D*. *vexillum* (New Zealand), but not in the native range (Japan). Recent studies of invasive populations have found DNA methylation differences between environments that may play a role in adaptation to different habitats^49,76,77^. In the present study, comparisons of DNA methylation patterns between sites with similar or different environmental stress (temperature and salinity) scores indicate that DNA methylation patterns are not driven by environmental variations alone. However, it remains possible that environmental stress is an important driver of epigenetic structure in the invasive New Zealand population. Random DNA methylation changes have been shown to be more likely following exposure to environmental stressors^78^. Invasive populations may accumulate novel epigenetic marks more quickly than populations in the native range if the invasion process can be considered a stress^51^, especially if combined with additional environmental challenges^60^. When unpredictable environmental conditions are encountered, epigenetically variable offspring will be favored, serving as a ‘bet-hedging’ strategy if some individuals are suited to the invaded habitat^79–82^. Additionally, it is possible that the founding genotypes that successfully established in New Zealand exhibit a higher propensity for methylation plasticity due to environmental filtering. Site-specific differentiation in the introduced populations could be due to the introduction and spread of epigenetic variants to different sites which, like genetic drift, is likely to have a large effect in small populations. However, these processes rely on a level of stability and heritability of methylation. It is not yet clear how much DNA methylation variation underlies selectable phenotypic variation, or how it responds to evolutionary forces^83^.

While we found DNA methylation differences between colonies from New Zealand and Japan, and between sites within New Zealand, these were associated with significant genetic (NML) differences. Invasive populations can include a different admixture of genes or genotypes compared to those in the native range, due to founder effects or multiple introductions from different source populations. Therefore, DNA methylation differences might reflect genetic differences between the native and introduced populations and between sites within the introduced range. Invasive populations can also be exposed to variable selection pressures across the newly colonised range, and have a faster evolutionary response to selection^79^. Models have shown that during early adaptation to new environments, epigenetic shifts could provide a “stepping stone”, facilitating the persistence of populations and allowing for the accumulation of neutral genetic variation^84^. Some epigenetic variation might become assimilated, meaning plastic responses or phenotypes are epigenetically encoded first, followed by genetic evolution^85,86^. Stress-induced reductions in DNA methylation can also affect genetic variation by increasing transposition events^87^. The results from this study cannot establish whether DNA methylation differences between sites are pure, obligatory or facilitated, but all three types are likely to play a role in range expansions^23,69^. Selection on epigenetic variation, in addition to genetic variation, can allow populations to adapt faster than selection on genetic variation alone^84^.

The introduced New Zealand population had lower methylation levels compared to populations in the native range. Reduced levels of DNA methylation have been suggested to be a common ‘signature of invasion’, increasing phenotypic plasticity during the expansive phase of invasion when it is most required^51,60^. However, the mechanistic link between levels of methylation, environmental stress, biological invasions and adaptation to new environments is not well understood. Few studies have reported methylation levels in aquatic invertebrates and, of those that have, it appears that increased genome-wide methylation is the most common response to environmental stress^47,88–91^. Evidence suggests that high levels of gene body methylation can reduce spurious transcription and transcriptional noise (variability of gene expression levels) and may be a defense mechanism to combat the damaging effects of environmental challenges^88,92^. In contrast, genes with reduced levels of germline DNA methylation have been linked with increased phenotypic plasticity via associations with alternative transcription start sites, exon skipping, alternative splicing and transient methylation^52,92,93^. Hypomethylation has also been associated with increased activity of transposable elements in several species, which can introduce phenotypic variation and may facilitate adaptation to new environments^87,94^. While stable and accurate gene expression could be beneficial within the native range of a species, where genetic diversity is generally high and environmental conditions are predictable from one generation to the next, following invasion, noisy or unstable genomes might be especially beneficial. If environmental stress is associated with higher levels of DNA methylation, it should be considered that lower levels of methylation might be associated with a reduction in stress. However, there is little evidence for this at present. One study has compared invasive populations of pygmy mussels (*Xenostrobus secures*) from two sites with apparently varying levels of environmental stress (an international port and a protected lagoon)^51^. It was expected that the population from the less stressful environment (the lagoon) would be less methylated, however it was found that methylation levels between sites were similar. In this case, it was suggested that the ‘invasive signature’ might be overriding any differences associated with the environment. It should be noted that while Type IV methylation (hypermethylation) is considered uninformative because it could be due to target absence, a large amount could also be due to methylation polymorphisms, so could also be important when comparing methylation levels in invasive and native populations.

## Conclusion

This is the first study investigating DNA methylation differences between closely related invertebrate clades, one of which has become invasive and one of which has not. We found further evidence for a ‘signature of invasion’ associated with a recent population expansion^51^, with introduced populations having distinct DNA methylation patterns and levels compared to populations in the native range. These results suggest that genetic diversity might facilitate the spread of invasive populations outside the native range, while DNA methylation might be especially important in introduced populations when genetic diversity is reduced. The MSAP method used in this study only allows for analysis of global genomic patterns, so there may be important functional methylation differences between the invasive and non-invasive clades that were not detected here. Our future research will focus on examining the functional properties and stability of DNA methylation changes in response to different environmental conditions, which will aid our understanding of ecological adaptation and evolutionary dynamics, including the epigenetic basis of invasiveness.

## Supplementary information

Supplementary Figures and Tables

## Data Availability

The datasets analysed during the current study are available from the corresponding author on reasonable request.
