# Author Correction: Epigenetic patterns associated with an ascidian invasion: a comparison of closely related clades in their native and introduced ranges

**DOI:** 10.1038/s41598-019-55750-2

**Published:** 2019-12-13

**Authors:** Nicola A. Hawes, Achira Amadoru, Louis A. Tremblay, Xavier Pochon, Brendon Dunphy, Andrew E. Fidler, Kirsty F. Smith

**Affiliations:** 1 Cawthron Institute, 98 Halifax Street East, Nelson, 7010 New Zealand; 2 Institute of Marine Science, University of Auckland, Private Bag 92019, Auckland, 1142 New Zealand; 3 School of Biological Sciences, University of Auckland, Private Bag 92019, Auckland, 1142 New Zealand

Correction to: *Scientific Report* 10.1038/s41598-019-49813-7, published online 03 October 2019

The original version of this Article contained a typographical error in the spelling of the author Achira Amadoru, which was incorrectly given as Achira Amadorou. This has now been corrected in the PDF and HTML versions of the Article.

