# Supplementary material for: Epigenetic patterns associated with an ascidian invasion: a comparison of closely related clades in their native and introduced ranges
Source: Sci Rep. 2019 Oct 3;9:14275. doi: 10.1038/s41598-019-49813-7 (PMC6776620; doi:10.1038/s41598-019-49813-7)
Supplement: Supplementary file 1 — Supplementary Figures and Tables [file 41598_2019_49813_MOESM1_ESM.pdf]

Epigenetic patterns associated with an ascidian invasion: a comparison of closely related clades in their native and introduced ranges

Nicola A. Hawes[1,2]*, Achira Amadoru[1], Louis A. Tremblay[1,3], Xavier Pochon[1,2], Brendon Dunphy[2,3], Andrew E. Fidler[2], Kirsty F. Smith[1]

Supplementary Figures and Tables

Supplementary Table S1. Analysis of molecular variance (AMOVA) and degrees of freedom (d.f.) for methylation-sensitive loci (MSL) within *Didemnum vexillum* colonies from sites with summative stress scores (temperature stress score + salinity stress score) of 3 (PelA, n = 9 (New Zealand), ShiA, n = 12 and ShiB, n = 2 (Japan)) and 4 (WhaA, n = 14 (New Zealand), IzuB, n = 7 and SagB, n = 12 (Japan)). Significant *p*-values are shown in bold ($p < 0.05$).

Source	d.f.	SSD	MSD	Variance	Phi_ST	*p*
**Stress score 3**						
among groups	2	927	463.5	22.87	0.06783	**0.0069**
within groups	20	6287	314.3	314.3		
Total	22	7214	327.9			
**Stress score 4**						
among groups	2	1315	657.4	32.32	0.09315	**<0.0001**
within groups	30	9439	314.6	314.6		
Total	32	10754	336.1			

Supplementary Table S2. Post hoc comparison of the proportion of methylated loci (global methylation level) and summative stress scores using Tukey's honest significance difference (HSD) test.

Summative stress scores	Estimate Std.	Error	z value	***Pr*(>|z|)**
3 – 2	0.03924	0.18199	0.216	0.996
4 – 2	0.03357	0.17136	0.196	0.997
6 – 2	-0.19203	0.22687	-0.846	0.829
4 – 3	-0.00567	0.14314	-0.04	1
6 – 3	-0.23127	0.20638	-1.121	0.671
6 – 4	-0.2256	0.19708	-1.145	0.656

Supplementary Table S3. Analysis of molecular variance (AMOVA) and degrees of freedom (d.f.) for methylation-sensitive loci (MSL) and non-methylated loci (NML) in *Didemnum vexillum* clade A colonies from different sites in New Zealand (PelA, WhaA, QueA) compared to clade A colonies from different sites in Japan (ShiA, IseA). Significant *p*-values are shown in bold ($p < 0.05$).

Population	Source	d.f.	SSD	MSD	Variance	Phi_ST	*p* value
**MSL**							
PelA - ShiA	Among groups	1	608.3	608.3	29.24	0.08681	**0.0043**
	Within groups	19	5844	307.6	307.6		
	Total	20	6452	322.6			
PelA - IseA	Among groups	1	281	281	10.82	0.04664	**0.0354**
	Within groups	11	2432	221.1	221.1		
	Total	12	2713	226.1			
WhaA - ShiA	Among groups	1	646.8	646.8	26.72	0.0814	**0.0013**
	Within groups	24	7237	301.5	301.5		
	Total	25	7884	315.3			
WhaA - IseA	Among groups	1	291.8	291.8	12.4	0.05462	**0.0296**
	Within groups	16	3435	214.7	214.7		
	Total	17	3726	219.2			
QueA - ShiA	Among groups	1	422.1	422.1	6.196	0.01718	0.1083
	Within groups	20	7090	354.5	354.5		
	Total	21	7512	357.7			
QueA - IseA	Among groups	1	360.4	360.4	11.03	0.03578	0.0671
	Within groups	12	3568	297.4	297.4		
	Total	13	3929	302.2			
**NML**							
PelA - ShiA	Among groups	1	57.33	57.33	3.061	0.1059	**0.0017**
	Within groups	19	491.1	25.85	25.85		
	Total	20	548.4	27.42			
PelA - IseA	Among groups	1	121.6	121.6	11.95	0.1774	**0.0165**
	Within groups	11	609.5	55.41	55.41		
	Total	12	731.1	60.92			
WhaA - ShiA	Among groups	1	101.6	101.6	4.387	0.08897	**0.0039**
	Within groups	24	1078	44.92	44.92		
	Total	25	1180	47.19			
WhaA - IseA	Among groups	1	175.5	175.5	11.09	0.09426	**0.045**
	Within groups	16	1705	106.5	106.5		
	Total	17	1880	110.6			
QueA - ShiA	Among groups	1	21.25	21.25	0.5034	0.03096	0.0553
	Within groups	20	315.1	15.76	15.76		
	Total	21	336.4	16.02			
QueA - IseA	Among groups	1	56.9	56.1	3.612	0.0906	**0.0264**
	Within groups	12	435.1	36.26	36.26		
	Total	13	492	492			

Supplementary Table S4. Analysis of molecular variance (AMOVA) and degrees of freedom (d.f.) for methylation-sensitive loci (MSL) and non-methylated loci (NML) in *Didemnum vexillum* clade A colonies from different sites in New Zealand (PelA, WhaA, QueA) compared to clade B colonies collected from different sites in Japan (ShiB, IseB, SagB, IzuB). Significant *p*-values are shown in bold ($p < 0.05$).

Sites	Source	d.f.	SSD	MSD	Variance	Phi_ST	*p*
**MSL**							
PelA - ShiB	Among groups	1	245.5	245.5	14.42	0.06776	0.0556
	Within groups	9	1785	198.3	198.3		
	Total	10	2031	203.1			
PelA - IseB	Among groups	1	319.9	319.9	36.5	0.1541	0.0577
	Within groups	9	1804	200.4	200.4		
	Total	10	2123	212.3			
PelA - IzuB	Among groups	1	718.5	718.5	59.13	0.1896	**<0.001**
	Within groups	14	3539	252.8	252.8		
	Total	15	4258	283.8			
PelA - SagB	Among groups	1	556.4	556.4	25.28	0.07858	**0.0013**
	Within groups	19	5632	296.4	296.4		
	Total	20	6188	309.4			
WhaA - ShiB	Among groups	1	237.7	237.7	10.42	0.04925	0.1142
	Within groups	14	2817	201.2	201.2		
	Total	15	3054	203.6			
WhaA - IseB	Among groups	1	278.6	278.6	21.17	0.09381	0.0615
	Within groups	14	2863	204.5	204.5		
	Total	15	3142	209.5			
WhaA - IzuB	Among groups	1	865.1	865.1	62.71	0.1831	**<0.001**
	Within groups	19	5316	279.8	279.8		
	Total	20	6182	309.1			
WhaA - SagB	Among groups	1	562.7	562.7	21.15	0.06809	**<0.001**
	Within groups	24	6946	289.4	289.4		
	Total	25	7508	300.3			
QueA - ShiB	Among groups	1	282.4	282.4	-0.2915	-0.00103	0.3885
	Within groups	10	2834	283.4	283.4		
	Total	11	3116	283.3			
QueA - IseB	Among groups	1	362.3	362.3	22.54	0.07278	0.11
	Within groups	10	2872	287.2	287.2		
	Total	11	3234	294			
QueA - IzuB	Among groups	1	608.4	608.4	35.11	0.09909	**0.0024**
	Within groups	15	4789	319.2	319.2		
	Total	16	5397	337.3			
QueA - SagB	Among groups	1	458.7	458.7	10.43	0.02936	**0.0288**
	Within groups	20	6897	344.9	344.9		
	Total	21	7356	350.3			

Supplementary Table S4 cont.

NML							
PelA - ShiB	Among groups	1	140.9	140.9	24.16	0.2808	**0.0356**
	Within groups	9	556.9	61.88	61.88		
	Total	10	697.8	69.78			
PelA - IseB	Among groups	1	222	222	50.39	0.469	**0.0195**
	Within groups	9	513.5	57.06	57.06		
	Total	10	735.5	73.55			
PelA - IzuB	Among groups	1	155.1	155.1	14.32	0.2528	**<0.001**
	Within groups	14	592.7	42.34	42.34		
	Total	15	747.8	49.85			
PelA - SagB	Among groups	1	59.98	59.98	3.091	0.0988	**<0.001**
	Within groups	19	535.6	28.19	28.19		
	Total	20	595.6	29.78			
WhaA - ShiB	Among groups	1	163.6	163.6	12.68	0.09617	0.1178
	Within groups	14	1668	119.2	119.2		
	Total	15	1832	122.1			
WhaA - IseB	Among groups	1	271.5	271.5	45.32	0.2865	**0.0233**
	Within groups	14	1580	112.9	112.9		
	Total	15	1852	123.4			
WhaA - IzuB	Among groups	1	114.5	114.5	7.935	0.164	**0.0011**
	Within groups	19	768.4	40.44	40.44		
	Total	20	882.9	44.14			
WhaA - SagB	Among groups	1	114	114	4.789	0.08421	**0.004**
	Within groups	24	1250	52.08	52.08		
	Total	25	1364	54.55			
QueA - ShiB	Among groups	1	63.65	63.65	5.022	0.0967	0.1206
	Within groups	10	469.1	46.91	46.91		
	Total	11	532.8	48.43			
QueA - IseB	Among groups	1	101.7	101.7	17.49	0.2873	**0.0168**
	Within groups	10	433.9	43.39	43.39		
	Total	11	535.6	48.69			
QueA - IzuB	Among groups	1	60.17	60.17	3.913	0.1228	**<0.001**
	Within groups	15	419.1	27.94	27.94		
	Total	16	479.3	29.96			
QueA - SagB	Among groups	1	34.39	34.39	1.531	0.07965	**<0.001**
	Within groups	20	353.8	17.69	17.69		
	Total	21	388.1	18.48			

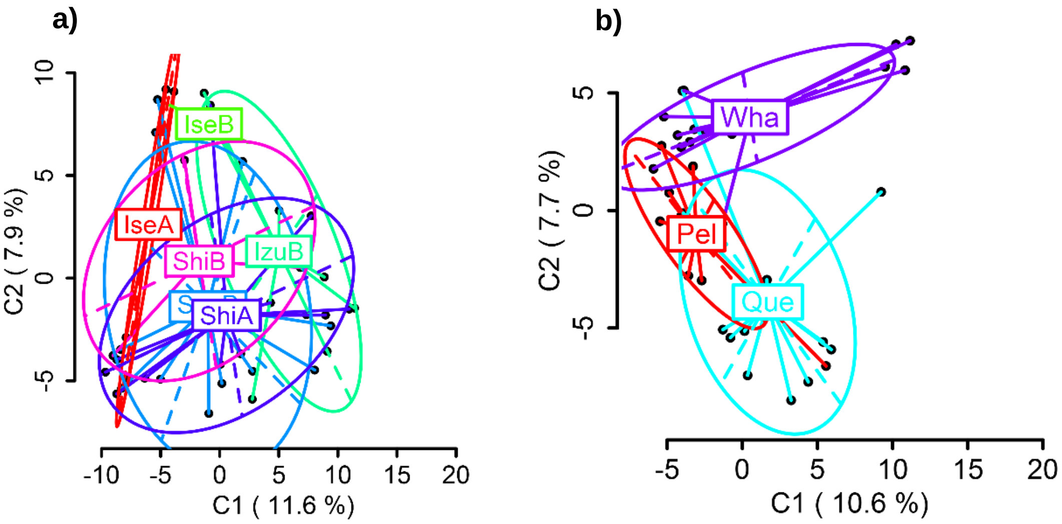

Supplementary Figure S5. Principal Coordinate Analysis (PCoA) of: **a)** non-significant methylation (MSL) differences in *Didemnum vexillum* colonies from different sites in Japan (JAA and JAB) and **b)** significant differences in *D. vexillum* colonies from different sites in New Zealand (NZA). NZA (n = 35), JAA (n = 16) and JAB (n = 23). The first two coordinates (C1 and C2) are shown with the percentage of variance explained by them. Points in each group cloud represent individuals from different groups. Site labels show the centroid for the points cloud in each group. Ellipses represent the average dispersion of those points around their centre (Pérez-Figueroa 2013). AMOVA tests for significant differences in methylation (MSL) are shown in Supplementary Table S6.

Supplementary Table S6. Analysis of molecular variance (AMOVA) and degrees of freedom (d.f.) for methylation-sensitive loci (MSL) and non-methylated loci (NML) in *Didemnum vexillum* colonies from different sites in New Zealand (NZA). Significant *p*-values are shown in bold ($p < 0.05$).

**Sites**	**Source**	**d.f.**	**SSD**	**MSD**	**Variance**	**Phi_ST**	***p* value**
**MSL**							
Que - Wha	Among groups	1	504.6	504.6	18.76	0.0668	**<0.001**
	Within groups	24	6291	262.1	262.1		
	Total	25	6795	271.8			
Pel - Wha	Among groups	1	354.4	354.4	11.28	0.04657	**0.019**
	Within groups	21	4849	230.9	230.9		
	Total	22	5203	236.5			
Pel - Que	Among groups	1	370.6	370.6	10.55	0.03871	**0.0135**
	Within groups	19	4979	262.1	262.1		
	Total	20	5350	267.5			
**NML**							
Que - Wha	Among groups	1	114.7	114.7	4.373	0.06987	**0.0257**
	Within groups	24	1397	58.21	58.21		
	Total	25	1512	60.47			
Pel - Wha	Among groups	1	118.2	118.2	5.388	0.08339	**0.0164**
	Within groups	21	1244	59.22	59.22		
	Total	22	1362	61.9			
Pel - Que	Among groups	1	50.07	50.07	1.907	0.05891	**<0.001**
	Within groups	19	578.7	30.46	30.46		
	Total	20	628.8	31.44			

Supplementary Table S7. Methylation susceptible loci (MSL) and non-methylated loci (NML) diversity in *Didemnum vexillum* clade A colonies from Japan (JAA), clade B colonies from Japan (JAB) and clade A colonies from New Zealand (NZA) estimated by the Shannon diversity index (I). Differences between MSL and NML diversity using the Wilcoxon Rank Sum test with continuity correction (W). Significant *p*-values are shown in bold ($p < 0.05$).

**Population**	**Loci**	**I**	**SD**	**W**	***p* value**
JAA	MSL	0.6248769	0.08002103	304067.5	**< 0.001**
	NML	0.4849764	0.1416978		
JAB	MSL	0.6129504	0.09454218	172270	**< 0.001**
	NML	0.3528194	0.135363		
NZA	MSL	0.5997667	0.09853787	499941	**< 0.001**
	NML	0.3665219	0.1590017		

Supplementary Table S8. Analysis of variance (ANOVA) and degrees of freedom (d.f.) comparing methylation susceptible loci (MSL) and non-methylated loci (NML) diversity estimated by the Shannon diversity index in *Didemnum vexillum* clade A colonies from Japan (JAA), clade B colonies from Japan (JAB) and clade A colonies from New Zealand (NZA). Significant *p*-values are shown in bold ($p < 0.05$).

**Source**	**d.f.**	**SS**	**Variance**	**F**	***p* value**
**MSL**					
Between groups	2	0.0074	0.0037	0.4201	0.6586
Within groups	71	0.6228	0.0088		
Total	73	0.6302			
**NML**					
Between groups	2	0.1951	0.0975	4.4284	**0.0154**
Within groups	71	1.5639	0.022		
Total	73	1.7589			

Supplementary Table S9. Post hoc comparison of non-methylated loci (NML) diversity estimated by the Shannon diversity index compared among *Didemnum vexillum* populations (JAA, JAB, and NZA) using Tukey's honest significance difference (HSD) test. Significant *p*-values are shown in bold ($p < 0.05$).

**Populations**	**Diff**	**Lwr**	**upr**	***p* adj**
JAA - JAB	-0.1322	-0.2478	-0.0165	**0.0212**
JAA - NZA	-0.2257	-0.2257	-0.0112	**0.0269**
JAB - NZA	0.0137	-0.0817	0.1091	0.9369

Supplementary Table S10. Quasibinomial generalized linear model for the proportion of methylated loci (Type II + III/Type I + II + III) in *Didemnum vexillum* clade A colonies from Japan (JAA), clade B colonies from Japan (JAB) and clade A colonies from New Zealand (NZA). Significant *p*-values are shown in bold ($p < 0.05$).

**Population**	**Estimate**	**Std. Error**	**t value**	***Pr*(>|t|)**
JAA	-0.01382	0.11389	-0.121	0.9037
JAB	0.15637	0.14895	1.05	0.2974
NZA	-0.31771	0.14032	-2.264	**0.0266**

References

Pérez-Figueroa A (2013) msap: a tool for the statistical analysis of methylation-sensitive amplified polymorphism data. Molecular ecology resources 13:522-527
